# Quality or quantity of life? Treatment priorities in older adults with cancer in the community

**DOI:** 10.1093/oncolo/oyaf261

**Published:** 2025-08-20

**Authors:** Gabriel Aleixo, Julianne Ani, Ramy Sedhom

**Affiliations:** Department of Medicine, Perelman School of Medicine, University of Pennsylvania, Philadelphia, PA 19104, United States; Penn Medicine Princeton Health, Penn Medicine, Plainsboro, NJ 08540, United States; Department of Medicine, Perelman School of Medicine, University of Pennsylvania, Philadelphia, PA 19104, United States; Leonard Davis Institute of Health Economics, University of Pennsylvania, Philadelphia, PA 19104, United States; Penn Center for Cancer Care Innovation, Abramson Cancer Center, Penn Medicine, Philadelphia, PA 19104, United States

**Keywords:** geriatric oncology, quality of life, patient preference, incurable cancer community oncology

## Abstract

Older adults with cancer often face decisions about prioritizing quality of life, survival, or both. In this prospective study of 181 patients aged ≥65 at a community cancer center, patients completed a geriatric assessment (GA) that included a validated trade-off question: “Maintaining my quality of life is more important to me than living longer.” Preferences were categorized as prioritizing quality of life, quantity of life, or both. Older age (OR 1.06; *P* = .04) and female sex (Odds Ratio 2.82; *P* = .01) were associated with prioritizing quality of life. Functional, cognitive, or psychosocial impairments were not. Prioritizing quality of life was not associated with worse overall survival (Hazard Ratio 1.06; *P* = .89). Receipt of standard treatment improved survival (HR 0.38; *P* = .04), while Performance status 3-4 predicted worse outcomes. These findings challenge assumptions that prioritizing quality of life compromises survival and support the integration of GA and values-based discussions into cancer care.

## Introduction

Patients with cancer are often faced with difficult decisions about whether to prioritize quality of life, prolong survival, or find a balance between the 2. While younger, married individuals tend to prioritize survival, preferences vary widely, and few studies have examined this question specifically among older adults.^1–3^ Older adults often hold distinct care preferences shaped by life experience, comorbidities, and functional status.^4^ Yet their voices remain underrepresented in clinical trials, and their values are not routinely incorporated into treatment planning. In this study, we explored whether older adults with cancer prioritize quality over quantity of life, what factors influence this decision, and whether such preferences impact survival outcomes.

## Methods

### Population

This single-center prospective cohort study was conducted at a Penn Medicine–affiliated community hospital. Patients aged 65 years or older with a confirmed cancer diagnosis completed a comprehensive geriatric assessment (GA) at the time of diagnosis. The GA included validated assessments of functional status, social support, and cognitive function. As part of this standard process, patients also responded to a validated trade-off question: “Maintaining my quality of life is more important to me than living longer.” Responses were categorized into 3 groups: prioritizing quality of life, prioritizing length of life, or valuing both equally.^5^

### Statistical analysis

Descriptive statistics were used to summarize patient characteristics. For analytical purposes, patients who prioritized length of life and those who selected both were grouped together, to allow for greater statistical power and contrast with those who prioritized quality of life alone. This approach aimed to isolate a more definitive preference, especially relevant at the time of diagnosis.

Univariate and multivariable logistic regression were used to assess associations between patient characteristics and care preferences. The multivariable model included age, sex, functional impairment, nutritional impairment, social support, cognitive/psychological impairment, race, marital status, whether the cancer was potentially curable, and ECOG performance status. Overall survival was analyzed using Kaplan-Meier curves and Cox regression.

## Results

Between December 2023 and February 2025, 181 patients were analyzed. Table 1 summarizes patients’ characteristics. Those prioritizing quality of life were slightly older (median age 80 vs 77 years) and more frequently had stage III disease (26% vs 9%). Functional impairment was present in over 75% of all patients, and 10% had ECOG 3-4 status. Radiation therapy was more common among those prioritizing quality of life (70% vs 51%, *P* = .01).

**Table 1.** Patients’ characteristics.

Factor	Quality and length or only length or unknown	Quality of life as solo priority	*P*-value
**Number of patients**	99	82	
**Age, median (IQR)**	77 (74, 83)	80 (75, 86)	.04
**Sex**			
**Male**	44 (44%)	22 (27%)	.01
**Female**	55 (56%)	60 (73%)	
**White**	80 (81%)	66 (80%)	.87
**Non-Hispanic**	97 (98%)	78 (95%)	.34
**Marital**			.01
**Divorced**	9 (9%)	8 (10%)	
**Married**	59 (60%)	34 (41%)	
**Single**	13 (13%)	7 (9%)	
**Widowed**	18 (18%)	33 (40%)	
**Stage**			.02
**Unknown**	16 (16%)	14 (17%)	
**Stage I**	8 (8%)	7 (9%)	
**Stage II**	13 (13%)	4 (5%)	
**Stage III**	9 (9%)	21 (26%)	
**Stage IV**	53 (54%)	36 (44%)	
**Curable disease**	25 (27%)	25 (33%)	.45
**Diagnosis**			.35
**Lung**	15 (15%)	11 (13%)	
**GI**	15 (15%)	18 (22%)	
**Breast**	25 (25%)	12 (15%)	
**Melanoma/skin cancers**	4 (4%)	1 (1%)	
**Genito urinary**	10 (10%)	9 (11%)	
**Gynecological**	11 (11%)	12 (15%)	
**Pancreas/cholangiocarcinoma/hepatocellular**	12 (12%)	8 (10%)	
**Hematological cancers**	7 (7%)	9 (11%)	
**Unknown/sarcoma**	0 (0%)	2 (2%)	
**Functional impairment**	75 (76%)	66 (80%)	.45
**Nutrition impairment**	57 (58%)	46 (56%)	.84
**Poor social support**	36 (36%)	30 (37%)	.98
**Anxiety**	17 (17%)	8 (10%)	.15
**Depression**	22 (22%)	18 (22%)	.97
**Cognitive impairment**	7 (7%)	4 (5%)	.54
**Spiritual**	70 (71%)	57 (70%)	.78
**Advanced directive**	47 (47%)	38 (45%)	.53
**ECOG**			
**0-2**	89 (90%)	73 (89%)	.85
**3-4**	10 (10%)	9 (11%)	
**Physical therapy consult**	63 (64%)	48 (59%)	.48
**Nutrition consult**	58 (59%)	42 (51%)	.32
**Psychology consult**	20 (20%)	15 (18%)	.75
**Chaplain consult**	22 (22%)	10 (12%)	.08
**Social work consult**	59 (60%)	49 (60%)	.98
**Palliative care consult**	29 (29%)	32 (39%)	.17
**Geriatric navigator consult**	50 (51%)	43 (52%)	.8
**Chemotherapy or target therapy used**	82 (91%)	60 (82%)	.09
**Radiation**	45 (51%)	51 (70%)	.01
**Surgery**	34 (40%)	34 (46%)	.41
**Care**			
**Standard of care**	19 (26%)	19 (30%)	
**De-escalation**	46 (64%)	38 (59%)	
**Escalation**	7 (10%)	7 (11%)	.86
**Hospice**	21 (22%)	21 (26%)	.58
**Hospitalization**	33 (33%)	25 (30%)	.68
**Intensive care unit use**	5 (5%)	3 (4%)	.65
**Hospitalization in the last 30 days**	5 (31%)	3 (19%)	.41

In prespecified multivariable analysis, older age (OR 1.05, 95% CI, 1.00-1.09, *P* = .034) and female sex (OR 2.82, 95% CI, 1.35-5.88, *P* = .006) were significantly associated with prioritizing quality of life. Functional impairment (OR 1.48, *P* = .356), poor social support (OR 1.31, *P* = .487), and cognitive/psychological impairment (OR 0.54, *P* = .079) were not significantly associated with this preference (Table 2).

**Table 2.** Multivariable logistic regression: factors associated with prioritizing quality of life.

Variable	Odds ratio	95% CI	*P*-value
**Age (per year increase)**	**1.06**	**1.00-1.11**	**.04**
**Female sex**	**2.82**	**1.35-5.88**	**.01**
**Functional impairment**	1.47	0.39-5.46	.57
**Nutritional impairment**	1.24	0.54-2.78	.62
**Poor social support**	1.31	0.27-6.61	.74
**Anxiety/depression/cognitive impairment**	0.54	0.27-1.07	.08
**Race: White vs Black**	1.19	0.37-3.87	.77
**Marital status: Not married vs married**	1.08	0.55-2.08	.81
**Cancer considered curable**	1.16	0.53-2.55	.705
**ECOG performance status (per unit)**	**0.39**	**0.27-0.61**	**<.001**

Bold value is statistically significant.

In the full cohort, prioritizing quality of life was not associated with worse overall survival (HR 1.05, *P* = .89, Figure S1). ECOG 3-4 status was associated with worse survival (HR 2.51, *P* = .04), and receipt of standard treatment was associated with improved survival (HR 0.38, *P* = .04) (Table S1).

Among patients with incurable disease, survival was not significantly associated with care preferences (HR 1.03, *P* = .92), age (HR 1.05, *P* = .11), ECOG 3-4 vs 0-2 (HR 2.26, *P* = .08), or receipt of standard treatment (HR 0.61, *P* = .28).

## Discussion

This study offers insights into how older adults with cancer prioritize treatment goals and how those preferences relate to outcomes. Notably, prioritizing quality of life did not compromise overall survival, challenging assumptions that less aggressive care necessarily leads to worse outcomes in older adults. Female sex and older age were associated with prioritizing quality of life, echoing prior findings.^4^

Contrary to earlier studies, we found no significant association between functional impairment and preference for quality of life.^6^ This may reflect the sensitivity of standardized GA tools, which capture impairments that may be underrecognized in routine oncology practice. More than 75% of our cohort was functionally impaired—a finding that underscores the importance of GA in older adults, as emphasized by American Society of Clinical Oncology and International Society of Geriatric Oncology guidelines.^7^^,^^8^ Our findings suggest a disconnect between patients’ stated preferences and the treatments they received, reflecting the complex influence of patient, provider, and system-level factors. The lack of association between patient vulnerabilities and care goals may stem from emotional responses at diagnosis or the challenge of making value-based decisions under stress. These results underscore the need for structured, ongoing shared decision-making—especially in older adults—using geriatric communication tools such as the “Best Case/Worst Case” framework to support goal-aligned care throughout the cancer trajectory.^9^

Among patients with incurable disease, survival did not differ by treatment preference, ECOG status, or receipt of standard therapy. While this may be due to sample size limitations, it also reflects known limitations of ECOG performance status as a predictor of treatment tolerance and outcomes in older adults. Prior studies have shown that GA domains often outperform ECOG in predicting toxicity and mortality in this population.^10^

Strengths of this study include its prospective design, use of validated assessments, and focus on older adults—a group often excluded from decision-making studies. Limitations include the single-center design, limited sample size, reliance on a single time point for assessing care preferences, binary classification of complex values, and insufficient follow-up to assess median survival. Future work will explore how preferences evolve over time and how to better align treatments with patient values.

## Conclusion

Our findings challenge conventional assumptions in geriatric oncology. Many older adults express a preference for quality of life, and doing so does not appear to negatively impact survival—even in the setting of incurable disease. These results highlight the importance of individualized, values-based care and the need for oncologists to actively engage patients in meaningful discussions about what matters most to them.

## Supplementary Material

oyaf261_Supplementary_Data

## Data Availability

The data supporting the findings of this study are available from the authors via email upon reasonable request and subject to evaluation.
