# Supplementary material for: Quality or quantity of life? Treatment priorities in older adults with cancer in the community
Source: Oncologist. 2025 Aug 20;30(9):oyaf261. doi: 10.1093/oncolo/oyaf261 (PMC12449121; doi:10.1093/oncolo/oyaf261)
Supplement: oyaf261_Supplementary_Data [file oyaf261_supplementary_data.zip › Supplemental figure S1.docx]

**Supplemental figure S1:** Kaplan Meier graph of survival - full cohort


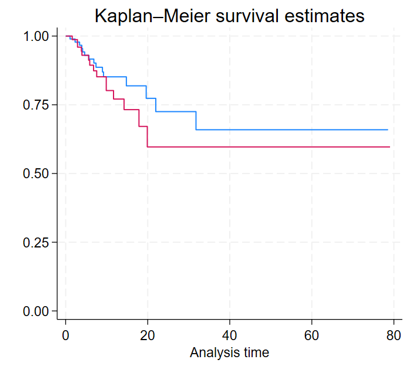


In the full cohort, prioritizing quality of life was not associated with overall survival (HR 1.05, p=0.89). ECOG status was associated with worse survival (HR 2.51, p=0.04), and receipt of standard treatment was associated with improved survival (HR 0.38, p=0.04)

Red – prioritize quality of life

Blue- prioritizes quantity of life
