# Supplementary material for: Quality or quantity of life? Treatment priorities in older adults with cancer in the community
Source: Oncologist. 2025 Aug 20;30(9):oyaf261. doi: 10.1093/oncolo/oyaf261 (PMC12449121; doi:10.1093/oncolo/oyaf261)
Supplement: oyaf261_Supplementary_Data [file oyaf261_supplementary_data.zip › Supplemental table S1.docx]

**Supplemental table S1.** Multivariable Cox Proportional Hazards Models for Overall Survival

| **Variable** | **Full Cohort (HR, 95% CI)** | ***p*-value** | **Incurable Only (HR, 95% CI)** | ***p*-value** |
| --- | --- | --- | --- | --- |
| Prioritizes Quality of Life | 1.06 (0.49–2.30) | 0.89 | 1.04 (0.47–2.31) | 0.92 |
| Age (per year) | 1.02 (0.97–1.09) | 0.44 | 1.06 (0.99–1.13) | 0.11 |
| ECOG PS (per unit increase) | 2.51 (1.05–6.00) | 0.04 | 2.26 (0.90–5.70) | 0.08 |
| Standard Treatment | 0.38 (0.15–0.94) | 0.04 | 0.61 (0.25–1.50) | 0.28 |
